# Understanding exopolysaccharide byproduct formation in *Komagataella phaffii* fermentation processes for recombinant protein production

**DOI:** 10.1186/s12934-024-02403-3

**Published:** 2024-05-06

**Authors:** Thomas Steimann, Zoe Heite, Andrea Germer, Lars Mathias Blank, Jochen Büchs, Marcel Mann, Jørgen Barsett Magnus

**Affiliations:** 1 AVT-Biochemical Engineering, RWTH Aachen University, Forckenbeckstraße 51, 52074 Aachen, Germany; 2 iAMB-Institute of Applied Microbiology, RWTH Aachen University, Worringer Weg 1, 52074 Aachen, Germany

**Keywords:** Exopolysaccharide, *Pichia pastoris*, *Komagataella phaffii*, Stirred tank reactor, Recombinant protein production, Cell wall composition, Metabolic engineering

## Abstract

**Background:**

*Komagataella phaffii* (*Pichia pastoris*) has emerged as a common and robust biotechnological platform organism, to produce recombinant proteins and other bioproducts of commercial interest. Key advantage of *K. phaffii* is the secretion of recombinant proteins, coupled with a low host protein secretion. This facilitates downstream processing, resulting in high purity of the target protein. However, a significant but often overlooked aspect is the presence of an unknown polysaccharide impurity in the supernatant. Surprisingly, this impurity has received limited attention in the literature, and its presence and quantification are rarely addressed.

**Results:**

This study aims to quantify this exopolysaccharide in high cell density recombinant protein production processes and identify its origin. In stirred tank fed-batch fermentations with a maximal cell dry weight of 155 g/L, the polysaccharide concentration in the supernatant can reach up to 8.7 g/L. This level is similar to the achievable target protein concentration. Importantly, the results demonstrate that exopolysaccharide production is independent of the substrate and the protein production process itself. Instead, it is directly correlated with biomass formation and proportional to cell dry weight. Cell lysis can confidently be ruled out as the source of this exopolysaccharide in the culture medium. Furthermore, the polysaccharide secretion can be linked to a mutation in the *HOC1* gene, featured by all derivatives of strain NRRL Y-11430, leading to a characteristic thinner cell wall.

**Conclusions:**

This research sheds light on a previously disregarded aspect of *K. phaffii* fermentations, emphasizing the importance of monitoring and addressing the exopolysaccharide impurity in biotechnological applications, independent of the recombinant protein produced.

**Supplementary Information:**

The online version contains supplementary material available at 10.1186/s12934-024-02403-3.

## Background

*Komagataella phaffii (K. phaffii)*, formerly referred to as *Pichia pastoris*, has gained recognition for its remarkable capability to efficiently secrete recombinant proteins into the surrounding medium. This not only reduces downstream processing efforts but also ensures a high purity of the expressed proteins, making it a preferred system of choice for various industrial applications. Furthermore, a mutation in the *HOC1* gene, present in most commercially used *K. phaffii* strains, leads to a thinner cell wall, enhancing protein secretion [1]. Moreover, *K. phaffii* is characterized by low secretion of host proteins, also contributing to cost-effective protein purification [2, 3].

However, an unexplored aspect of this established system demanding careful consideration is the presence of extracellular polysaccharides. This goes in hand with challenges for the protein production process, as the exopolysaccharides (EPS) need to be separated from the target protein, leading to higher purification costs. The existence of EPS was first mentioned by Trimble et al*.* [4]. An analysis of the produced glycoprotein revealed the presence of 3–4 times more carbohydrates in the supernatant than expected. Furthermore, Denton et al*.* [5] detected substantial quantities of an undefined and unknown EPS, significantly hindering protein crystallization and crystallographic analysis. Additional remarks of the EPS existence can be found in the works of Vinogradov et al*.* [6] and Włodarczyk-Biegun et al*.* [7]. Here, it was noted that the EPS is mainly composed of mannose units. It is also worth mentioning that the presence of the EPS will remain undetected when applying protein quantification methods like PAGE or liquid UV detectors. Although EPS quantification remains a relatively underexplored area in the literature, Werten et al*.* [8] emphasize the significance of EPS as the major impurity in *K. phaffii* culture broths. From data presented by Denton et al*.* [5], a rough estimate of the relative EPS concentration leads to 0.05–0.06 g/g_CDW_ (gram per gram of cell dry weight). To our knowledge, no further quantification of EPS related to *K. phaffii* is available in the existing literature. EPS byproduct formation is not limited to *K. phaffii*. For instance, other organisms such as *Vibrio natriegens* can produce up to 157 mg/L EPS as a byproduct at a cell density of 28 g/L [9]. Moreover, microbial EPS with potential industrial applications include xanthan from *Xanthomonas campesteris*, succinoglycan from *Rhizobium*, bacterial alginates from *Azotobacter vinelandii* and pullulan from *Aureobasidium pullulans* [10]. For pullulan 22 g/L can be reached, for *Bacillus polymyxa*, concentrations as high as 54 g/L are achievable [11–13].

Moreover, the research in protein purification from cultivations broths containing polysaccharide impurities remains limited, with two notable methods being lectin affinity chromatography [14, 15] and protein precipitation with ammonium sulfate [7] or acetone [4]. For pullulan purification, melanin separation is performed through adsorption on activated charcoal [10].

This study explores the origin of the unidentified EPS in high cell density *K. phaffii* fermentations, examining possible factors influencing the formation of the EPS. Therefore, several fermentations were performed varying the used strain, the carbon source, and the induction with methanol. Biomass and EPS formation were measured over the fermentation time to determine the relative EPS concentration and establish a relation with the examined factors.

## Methods

### Strains

The following three strains were used for experiments: First, *K. phaffii* Mut^S^ BSYBG11 (BG11) obtained from Bisy GmbH (Hofstaetten a. d. Raab, Austria). This strain is a derivative of strain *K phaffii* NRRL Y-11430/CBS 7435 and has a mutation in the *HOC1* gene leading to a truncated version of Hoc1 [16]. Second, strain BG11 has been used as host for the genomic integration of an expression cassette for a recombinant structural protein under control of the P_*CAT*_ promotor, in the following designated as strain I. It resulted in five target protein expression cassettes in the yur1 locus. The original sequence of the native host protein was codon optimized for *K. phaffii* to increase product titers. The strain is deposited at DSMZ as DSM 33957 and available under reasonable request. Third, the wildtype (Mut^+^) strain *K. phaffii* MUCL 46514 (NRRL Y-7556; CBS 2612; NRRL YB-4290) obtained from BCCM, hereafter referred to as Y-7556 was included into the study. This strain has an intact *HOC1* gene. The strain genealogy can be found in Additional file 1: Fig. S1.

### Media

All chemicals applied for media preparation were of analytical grade and purchased from Carl Roth GmbH (Karlsruhe, Germany), if not stated differently.

For shake flask precultures, the *K. phaffii* strains were grown in mineral Syn6-MES medium [17]. The basic Syn6-MES medium consisted of 1.0 g/L KH_2_PO_4_, 7.66 g/L (NH_4_)_2_SO_4_, 3.3 g/L KCl, 3.0 g/L MgSO_4_ × 7H_2_O, 0.3 g/L NaCl, 39 g/L (0.2 M) 2-(*N*-morpholino)-ethanesulfonic acid, 4-morpholineethanesulfonic acid (MES). All medium components were dissolved in deionized water, the pH was adjusted to 6.0 with 1 M NaOH and the medium was sterilized via autoclaving (121 °C for 20 min). Prio to use, 940 mL basic medium was supplemented with 10 mL of 100 g/L CaCl_2_ (sterile filtered), 10 mL of a 100 × micro-elements stock solution (sterile filtered), 10 mL of a 100 × vitamin stock solution (sterile filtered), 10 mL of a 100 × trace-elements solution (sterile filtered), and 20 mL glucose stock solution prepared with a concentration of 500 g/L (autoclaved). The stock solutions had the following compositions: micro-element stock solution: 6.65 g/L EDTA (ethylenediamine tetraacetic acid disodium sulfate), 6.65 g/L (NH_4_)_2_Fe(SO_4_)_2_ × 6H_2_O, 0.55 g/L CuSO_4_ × 5H_2_O, 2 g/L ZnSO_4_ × 7H_2_O and 2.65 g/L MnSO_4_ × H_2_O. Vitamin stock solution: 0.04 g/L d-biotin and 13.35 g/L thiamine chloride. The d-biotin was dissolved in 10 mL of a (1:1) mixture of 2-propanol and deionized water. Thiamin chloride was dissolved separately in 90 mL deionized water. Afterwards, the two solutions were mixed. Trace element stock solution: 0.065 g/L NiSO_4_ × 6H_2_O, 0.065 g/L CoCl_2_ × 6H_2_O, 0.065 g/L H_3_BO_3_, 0.065 g/L KI, and 0.065 g/L Na_2_MoO_4_ × 2H_2_O.

For stirred tank bioreactor cultivations a medium proposed by Hyka et al*.* [18] was prepared. The basic medium consists of 7.23 g/L H_3_PO_4_, 0.64 g/L KOH, 0.17 g/L CaSO_4_ × 2H_2_O, 2.86 g/L K_2_SO_4_, 2.3 g/L MgSO_4_ × 7H_2_O and 0.1 mL/L polypropylene glycol (PPG). The basic medium solution was sterilized via autoclaving (121 °C for 20 min) and a 650 g/L glucose or glycerol stock solution was added to a final concentration of 5 or 40 g/L. The medium was supplemented with 0.62 mL/L vitamin stock solution from Syn6-MES medium and 0.74 mL/L filter sterilized modified trace element solution PTM1. It consists of 3.84 g/L CuSO_4_ × 5H_2_O, 0.08 g/L NaI, 3 g/L MnSO_4_ × H_2_O, 0.2 g/L Na_2_MoO_4_ × 2H_2_O, 0.02 g/L H_3_BO_3_, 0.92 g/L CoCl_2_ × 6H_2_O, 20 g/L ZnCl_2_, 65 g/L FeSO_4_ × 7H_2_O and 5 mL/L 69 wt.% H_2_SO_4_. The medium components were dissolved in deionized water. The pH of the medium was titrated to 6.0 using ammonia solution (30 vol.%). Production was induced with 1 vol.% methanol (purity > 99.5%), if not stated otherwise.

The feed for bioreactor cultivations consisted of a 650 g/L glucose solution or 650 g/L glycerol solution or pure methanol (purity > 99.5%). The feed was supplemented with 12 mL/L PTM1 and 10 mL/L vitamin stock solution from Syn6-MES medium. After methanol induction of the bioreactor with 1 vol.%, the feed was also supplemented with 70 mL/L methanol. For non-induced fermentations, deionized water was added instead.

### Preculture

For bioreactor cultivations, a preculture was grown in four unbaffled 250 mL shake flasks with a filling volume of 10 mL. The flasks were inoculated with 100 µL glycerol stock cell suspension stored at − 80 °C (optical density measured at 600 nm OD_600_ = 5) and cultivated for 18 h in a temperature-controlled hood (Climo-Shaker ISF1-X, Kuhner, Birsfelden, Switzerland) at 30 °C with a shaking frequency of 350 rpm and a shaking diameter of 50 mm.

### Main culture

Fermentations were performed in a 2 L Sartorius BIOSTAT^®^ stirred tank reactor (Sartorius, Göttingen, Germany) equipped with 4 baffles and two 6-bladed Rushton turbines (58 mm diameter and 11 mm height) mounted at heights of 30 mm and 90 mm from the bottom. A peristaltic pump (101 U/R, Watson-Marlow Pump Group, Falmouth, UK) was used for the feed. Cultivation was started in batch mode after inoculation to a starting OD_600_ of 0.2. Fermentation experiments were performed with an initial filling volume of 900 mL. If not stated otherwise, after glucose depletion (spike in the dissolved oxygen tension) at ${t}_{1}$, the first feed was started as a pre-programmed carbon limiting exponential feed with a pre-set growth rate µ_SET_ of 0.2 1/h to further increase biomass concentration at a growth rate near the maximal growth rate of the cells. A maximal growth rate of 0.25 1/h is given in literature [19]. The feeding rate F_1_ is calculated applying the formula for a set constant growth rate given by Looser et al*.* [20] and in Eq. (1) with µ_SET_ the pre-set growth rate, Y_X/S_ the biomass yield, m_S_ the maintenance coefficient, V_0_ the filling volume and X_0_ the biomass concentration at the start of the feed and S_F_ the carbon concentration of the feed.

1 $$F\left(t\right)={\left(\frac{{\mu }_{SET}}{{Y}_{X/S}}+{m}_{S}\right)\cdot \frac{{V}_{0}\cdot {X}_{0}}{{S}_{F}}} \cdot \, e^{{\mu }_{SET} \left({t-t}_{0}\right)}.$$

Based on previous experiments, a biomass yield Y_X/S_ of 0.57 g/g and a maintenance coefficient m_S_ of 0.019 g/g/h is used for feed calculation. These values are in good accordance with literature [19, 21, 22]. The applied feed F_1_ is given in Eq. (2).

2 $${F}_{1}=11\frac{mL}{h} \cdot e^{0.2 {h}^{-1} \left({t-t}_{1}\right)}.$$

After 5 h, at ${t}_{2}$, the cells were induced with 1 vol.% methanol and the feeding rate reduced to F_2_ to reduce the growth rate µ_SET_ to 0.05 1/h. The feed rate is given in Eq. (3). For non-induced fermentations, 1 vol% deionized water was added instead of methanol for induction.

3 $${F}_{2}=7.5 {\frac{mL}{h}} \cdot e^{0.05 {h}^{-1} \left({t-t}_{2}\right)}.$$

The temperature was controlled at 28 °C, pH (EasyFerm Plus K8 225, Hamilton, Hoechst, Germany) at 6.0 with ammonia solution (30 vol.%), dissolved oxygen tension (VisiFermTM DO 225 pO_2_ sensor, Hamilton, Hoechst, Germany) at 30% air saturation by cascade control of stirring rate (500–1500 rpm) and aeration rate (1–3 sL/min).

### Offline analysis

Samples were taken for offline analysis on regular time intervals. The OD_600_, cell dry weight (CDW), target protein concentration and EPS concentration were determined. OD_600_ was measured using a Genesys 20 photometer (Thermo Scientific, Darmstadt, Germany). Samples were diluted with 0.9% (w/v) NaCl, if necessary. Samples were centrifuged at 18,000 rcf for 10 min and the supernatant was filtered with a 0.2 µm cut-off filter (Millipore-Sigma, Burlington, USA). Protein concentration was determined using size exclusion chromatography (GPC EcoSEC, Tosoh Bioscience GmbH, Stuttgart, Germany) equipped with 3 PROTEEMA columns (PSS Polymer, Mainz, Germany) and a UV detector (214 nm). The mobile phase consisted of 0.2 M phosphate buffer at a pH of 5.3 with a flow rate of 1 mL/min. The temperature was set to 40 °C. For calibration 2 g/L BSA (bovine serum albumin) was used.

### Determination of exopolysaccharide concentration

**Fig. 1:**
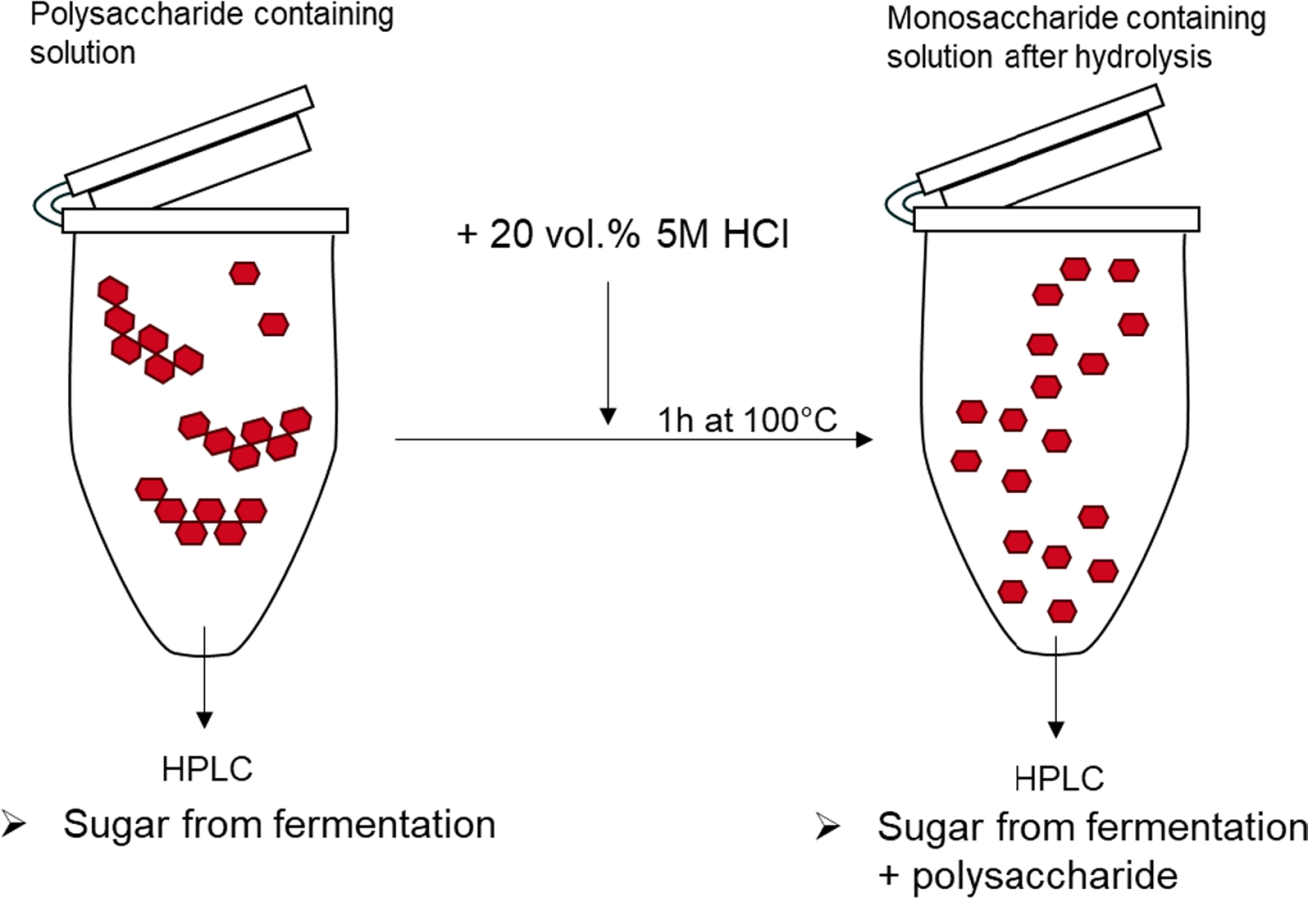
Method for polysaccharide detection in fermentation supernatant. Detection of sugar monomers via HPLC after acid hydrolysis of polysaccharide by addition of 20 vol.% of 5 M HCl and incubation at 100 °C for 1 h

To determine polysaccharide concentrations, 200 µL 5 M HCl solution was added to 1 mL sample. The samples were incubated for 1 h at 100 °C to hydrolyze the polysaccharide. The sugar concentration of the hydrolyzed sample was analyzed via HPLC and compared to the sugar concentration of the non-hydrolyzed sample. HPLC analysis was performed with a Thermo Fisher Ultimate 3000 (Thermo Fisher Scientific Inc., Waltham, USA), equipped with an ERC RefractoMax 520 RID (Shodex, Munich, Germany). Separation was performed with an Organic Acid (300 × 7.8 mm) column (Phenomenex, Torrance, USA), heated to 80 °C and a mobile phase of 25 mM H_2_SO_4_ running at 0.8 mL/min. The procedure is schematically depicted in Fig. 1. Blank and positive controls were always measured in parallel. The positive control consisted of a 4 g/L starch solution (Merck, Darmstadt, Germany). The results from the starch hydrolysis are shown in Additional file 1: Fig. S2.

EPS concentration is referenced to CDW. CDW is determined from OD using Eq. (4). Data for the correlation is given in Additional file 1: Fig. S3.

4 $$CDW= \frac{OD}{1.3} [g/L].$$

Statistical analysis tools (OriginPro 2022, OriginLab Corporation, Northampton, USA) were used to determine the significance of the data. Normal distribution was assumed, and inhomogeneity of variance was determined using Levene’s test. Therefore, a Welch’s ANOVA with a significance level of α = 0.05 was used to determine significant differences between experiments. Games-Howell pairwise comparisons were performed, to determine between which groups the difference was significant.

### Determination of cell lysis

Samples were diluted 1:1000 with 0.5 mol.% PBS buffer. The PBS buffer consisted of 137 mM NaCl, 2.7 mM KCl, 10 mM Na_2_HPO_4_ and 1.8 mM KH_2_PO_4_. Cell lysis was determined by impedance flow cytometry with Ampha Z_32_ (Amphasys, Root, Switzerland) at a frequency of 24 MHz immediately after sampling. The C chip (50 × 50 µm) was used for measurements. The vertical gate for lysed cells was set at a phase of 208°. For positive control, cell samples lysed for 20 min at 100 °C were used.

## Results and discussion

### Quantification of exopolysaccharide formation

**Fig. 2:**
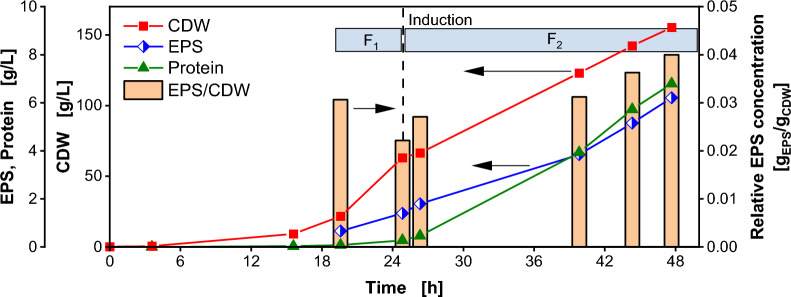
Fed-Batch fermentation of *Komagataella phaffii* recombinant protein producing strain I performed in a 2 L stirred tank reactor. After 25 h product formation is induced by methanol addition. Cell dry weight (red squares), EPS concentration in supernatant (blue diamonds), target protein in supernatant (green triangles) and relative EPS concentration determined by the ratio of exopolysaccharide (EPS) and cell dry weight (CDW) (orange bars) is depicted over time. Cultivation was performed with 40 g/L initial glucose concentration. Feeding solution consisted of 650 g/L glucose. Production was induced with 1 vol.% MeOH. 70 mL/L MeOH was added to the feeding solution after induction. Feeding rate F_1_ = 11 mL/h *exp(0.2 h^−1^ * t) from 20 h to 25 h. Feeding rate F_2_ = 7.5 mL/h *exp(0.05 h^−1^ * t) from 25 h to 48 h

In order to quantify exopolysaccharide (EPS) formation, a reference fed-batch fermentation was conducted in a 2 L stirred tank reactor with the *K. phaffii* strain I. The results are shown in Fig. 2. Offline samples were collected at various time points to determine EPS concentrations, target protein concentrations and cell dry weight (CDW). Additionally, the relative EPS concentration was calculated as the ratio of the EPS to CDW. The cultivation started as a batch with 40 g/L glucose. Upon glucose depletion after 20 h (Glucose concentration shown in Additional file 1: Fig. S4), the growth phase was extended for further 5 h with an exponentially increasing feed F_1_. Up to induction, the cells indeed exhibit exponential growth, as evident from the exponentially increasing CDW. Notably, the target protein concentration shows a minimal increase prior to induction. A possible explanation is basal expression, already shown for recombinant protein expression under the P_*CAT*_ promotor in *K. phaffii* under carbon limiting conditions prior to induction [23]. The P_*CAT*_ promotor has even been suggested as a derepressed promotor enabling inducible, methanol-free processes [24, 25]. After 25 h, the production of the target protein was induced by methanol addition and growth was reduced via the feed rate. Following induction, a notable increase in product formation is observed, ultimately reaching a final target protein titer of 6.8 g/L and a CDW concentration of 155 g/L after 48 h. The results are comparable to other *K. phaffii* expression systems, as reviewed by Cereghino and Cregg [26]. The EPS concentration increases during the fermentation proportional to CDW, as indicated by the constant relative EPS concentration ranging from 0.022 to 0.040 g_EPS_/g_CDW_. The total EPS concentration at the end of the fermentation reaches 6.2 g/L, consistent with the findings presented by Denton et al*.* [5]. There, a ratio of 0.05–0.06 g_EPS_/g_CDW_ can be estimated from the presented data. Compared to other microbial EPS byproduct producing systems, the concentration is relatively high. For *Vibrio natriegens*, EPS byproduct only reaches 0.005 g_EPS_/g_CDW_ [9].

**Fig. 3:**
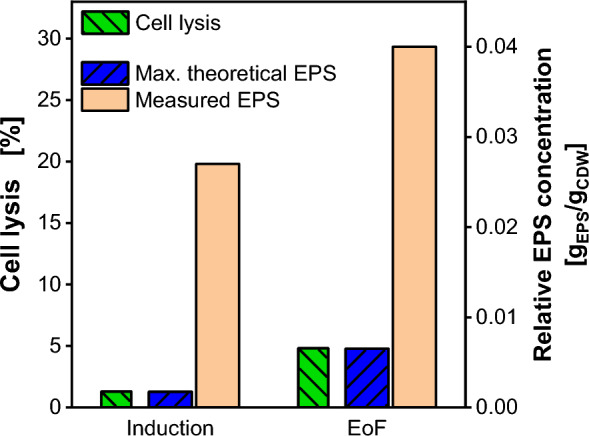
Cell lysis percentage during fermentation process and derived maximal theoretical relative exopolysaccharide concentration. Cell lysis determined by impedance flow cytometry. Results are compared to measured relative EPS concentration. Samples at induction time (25 h) and the end of fermentation (EoF, 48 h) for the process shown in Fig. 2 are compared. Cell distribution is shown in Additional file 1: Fig. S4. Maximal theoretical relative exopolysaccharide concentration calculated from Eq. (5)

A potential explanation for the presence of an EPS in the supernatant is cell lysis, considering that polysaccharides constitute a major component of yeast cell wall material. According to Roelofsen [27], the cell wall accounts for 20% (w/w) of the cell dry weight in *Saccharomyces cerevisiae* (*S. cerevisiae*), with 68% (w/w) of it being polysaccharides. Therefore, assuming a similar composition of the cell wall for *K. phaffii*, the maximal EPS concentration per CDW resulting from lysed cells EPS_lysed_ is theoretically 0.136 g_EPS_/g_CDW,lysed_. For two samples from the fermentation depicted in Fig. 2, cell lysis was determined. The results are presented in Fig. 3 (cell lysis distribution is shown in Additional file 1: Fig. S5). In green, the cell lysis percentage is shown after methanol induction (Fig. 3: Induction after 25 h) and at the end of the fermentation (Fig. 3: EoF after 48 h). The highest cell lysis percentage x_lysed_ of 4.8% is measured after 48 h at the end of the fermentation (EoF). In blue, the maximal theoretical relative EPS concentration originated from cell lysis EPS_th,max_ is plotted. This is calculated considering the maximal polysaccharide amount of the cells with Eq. (5):

5 $${EPS}_{th,max}={EPS}_{lysed}\cdot {x}_{lysed.}$$

For highest measured cell lysis after 48 h, an EPS concentration of 0.0065 g_EPS_/g_CDW_ can be calculated (Eq. 6).

6 $${EPS}_{th,max}=0.136 \frac{{g}_{EPS}}{{g}_{CDW,lysed}}\cdot 4.8\% \frac{{g}_{CDW,lysed}}{{g}_{CDW}}= 0.0065 \frac{{g}_{EPS}}{{g}_{CDW}}.$$

In orange, the measured relative EPS concentration from Fig. 2 is depicted. Clearly, the EPS concentration explainable through cell lysis, is significantly lower than the measured EPS in the samples. Cell lysis can only account for 16% of the measured EPS. Therefore, cell lysis cannot be the cause for the high EPS concentration.

### Influence of process conditions on exopolysaccharide formation

**Fig. 4:**
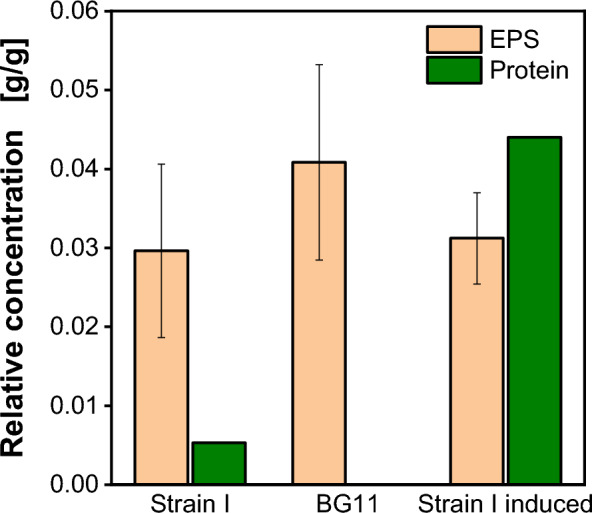
Relative exopolysaccharide and protein concentration determined in 2 L stirred tank fermentations with strain I with 40 g/L glucose batch and glucose feed (not induced), BG11 strain with 40 g/L glucose batch and glucose feed (not induced) and strain I in a methanol (1 vol.%) induced process with 40 g/L glucose batch and mixed glucose/methanol feed. Relative EPS concentration averaged through fermentation time. Relative protein concentration determined at the end of the fermentation

The only possible alternative explanation is the production and secretion of EPS by *K. phaffii*. However, the causative relation between target protein production and EPS formation remains unclear. In order to understand this, two additional fermentations were conducted in 2 L stirred tank reactors: first, using strain I without inducing product formation, and second, utilizing the host strain BG11 without the expression cassette. Measurements of protein concentration, EPS concentration and CDW were conducted throughout the fermentation. In Fig. 4, protein concentration and EPS concentration from all three fermentations were plotted relative to the CDW. The protein concentration exhibits a substantial reduction in non-induced processes (Strain I and BG11) compared to the reference fermentation (Strain I induced). This is expected, as protein production is induced by methanol. However, some basal expression of the target protein is observed, as is the case in the reference fermentation (Fig. 2) before methanol induction.

The relative EPS concentration was averaged over the fermentation time and ranges between 0.03 and 0.04 g_EPS_/g_CDW_. Notably, statistical comparison of the data reveals no significant differences in relative EPS concentration among the three cultivations. Therefore, neither the integration of protein expression cassettes nor methanol induction appears to induce EPS formation in *K. phaffii*. Moreover, it is evident that the polysaccharide is not covalently linked to the expressed recombinant protein, as its secretion occurs independently of protein expression. This observation aligns with the findings of O'Leary et al*.* [15], who similarly concluded that the polysaccharide is not covalently linked to the expressed protein. It cannot be excluded, that the EPS is partially linked to native secreted background proteins.

**Fig. 5:**
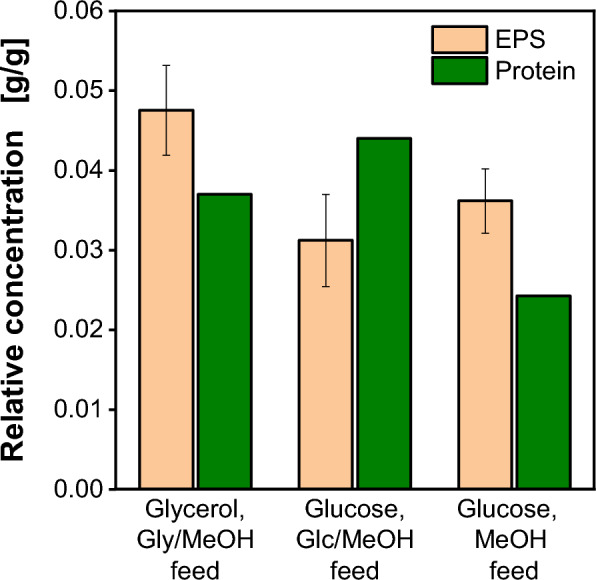
Relative exopolysaccharide and protein concentration determined in 2 L stirred tank fermentations with strain I with 40 g/L glycerol batch and mixed glycerol/methanol feed, 40 g/L glucose batch and mixed glucose/methanol feed and 40 g/L glucose batch and pure methanol feed. Production is induced with 1 vol.% MeOH. Relative EPS concentration averaged over fermentation time. Relative protein concentration determined at the end of the fermentation

To exclude the carbon source as a cause for EPS formation during fermentation, a comparison was made between fermentations using glycerol, sole methanol, and the reference fermentation employing glucose. Relative EPS and protein concentrations for these fermentations are presented in Fig. 5. The relative EPS concentration was averaged over the fermentation time. The fermentation with glycerol involved a glycerol/methanol feed to induce product formation. The fermentation with a pure methanol feed utilized methanol as the sole carbon source, after the initial batch phase with 40 g/L glucose. In this case, the feeding rate was reduced to F_2, MeOH_ (Eq. 7) due to the lower growth rate on pure methanol for Mut^S^ strains [28, 29].

7 $${F}_{2,MeOH}=0.5\frac{mL}{h} \cdot e^{0.015 {h}^{-1} \left({t-t}_{2}\right)}.$$

The resulting relative EPS concentrations of 0.03–0.05 g_EPS_/g_CDW_ exhibit no significant differences across the three fermentations, leading to the conclusion that the chosen carbon sources do not trigger or influence EPS formation in *K. phaffii*. Furthermore, the relative EPS concentration reaches the same level as seen in Fig. 4 (0.03–0.04 g_EPS_/g_CDW_), confirming that EPS formation is only related to biomass concentration and independent of the used carbon source and methanol induction. The influence of further fermentation conditions i.e. temperature, pH or dissolved oxygen on EPS formation is still to be assessed. Concerning product formation, the highest relative product concentration is achieved with glucose as the main carbon source and a glucose/methanol feed.

### Influence of strain background on exopolysaccharide formation

**Fig. 6:**
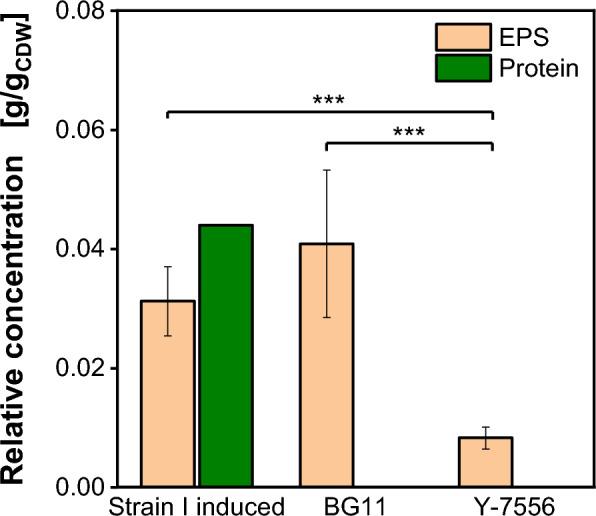
Relative exopolysaccharide and protein concentration determined in 2 L stirred tank fermentations with (**a**) strain I in a methanol induced process with 40 g/L glucose batch and mixed glucose/methanol feed, (**b**) strain BG11 in a non-induced process with 40 g/L glucose batch and glucose feed and (**c**) strain Y-7556 in a non-induced process with 40 g/L glucose batch and glucose feed. Relative EPS concentration averaged through fermentation time. Relative protein concentration determined at the end of the fermentation. Significant differences marked (*** for p < 0.001)

The presented results show strong evidence, that the EPS formation is independent of the recombinant protein produced in this study, methanol induction or carbon source. It cannot be ruled out, that other recombinant proteins may affect EPS formation, but it seems, that the EPS formation is purely proportional to biomass. The EPS concentration is always around 0.04 g_EPS_/g_CDW_ (Figs. 3, 4 and 5). Therefore, the strain's genetic background was investigated to generate an explanation for the EPS formation. The EPS formation of strain I and the host strain BG11 was compared to the genetic ancestor and true wildtype strain *K. phaffii* Y-7556 (*K. phaffii* strain genealogy can be found in Additional file 1: Fig. S1). The relative EPS and product concentrations for the three strains used in this study are shown in Fig. 6. Analogous to Figs. 4 and 5, the relative EPS concentration was determined over the course of the fermentation and averaged over all samples. The individual samples are included in the supplementary data (Additional file 1: Fig. S6). For the genetic ancestor strain Y-7556 (Fig. 6), a significant reduction of the EPS concentration of 76% from 0.03–0.04 g_EPS_/g_CDW_ to 0.0083 ± 0.0018 g_EPS_/g_CDW_ is measured, when compared to the BG11 strain (Fig. 6) and the strain I (Fig. 6). The remaining EPS could be explained by cell lysis. As mentioned before, a relative EPS concentration of 0.0065 g_EPS_/g_CDW_ could be plausibly explained by the measured cell lysis. The main difference between the ancestral strain Y-7556 and the BG11 strain (derivative of strain NRRL Y-11430) is a single base pair deletion in the *HOC1* gene in the latter strain, leading to a frameshift and a premature stop codon [16, 30]. The *HOC1* gene (homolog of *OCH1*) codes for an α-1,6-mannosyltransferase (UniProt IDF2QVW2), required for correct cell wall construction [16, 31]. It is known, that the *HOC1* mutation leads to a thinner, more permeable cell wall, enhancing protein secretion and transformation efficiency up to six times, compared to species without this frameshift [1]. This makes the *HOC1* mutation essential for strain performance.

Mannose containing polysaccharide is a main part of the cell wall material in yeast cells [27]. Interestingly, *Komagataella pastoris,* a close relative of *K. phaffii*, has recently been suggested as a potential source of mannose containing polysaccharides, which can be extracted from its cell wall [32]. Specifically in *S. cerevisiae*, a substantial portion of N-linked glycans on cell walls and periplasmic proteins undergo modification via mannose polysaccharide addition. The polysaccharide contains a central backbone comprising around 50 α-1,6-linked mannose units. Additionally, the backbone has several branches extending from it, mainly constituted of α-1,2-linked and α-1,3-linked mannoses. The structure of the polysaccharide and the involved enzymes for the construction are shown by Munro [33]. First, a single α-1,6-mannose is added to the cell wall proteins by the mannosyltransferase Och1 in the cis Golgi. This mannose is then extended to a mannose backbone by the sequential action of two mannan polymerase (M-Pol I and M-Pol II) protein complexes, one of them (M-Pol II) containing the mannosyltransferase encoded by *HOC1* [33, 34]. It is unclear, what the exact function of the Hoc1 protein is in the complex, but it may have a regulatory function of the mannan backbone length by providing an α-1,2-mannose cap [34]. Disruption of Hoc1 in *S. cerevisiae* has been observed to adversely affect both cell integrity and protein glycosylation [31].

The presented results show strong evidence that the frameshift in the *HOC1* gene in strain BG11 and strain I (Fig. 6) also leads to the strong EPS formation. The disfunction of the mannan polymerase M-Pol II caused by the malfunction of the Hoc1 protein may be the reason for the mannose polysaccharide secretion instead of the correct linkage to the cell wall. The polymerase may have a reduced selectivity and produce free mannan polymers, corresponding to the EPS. This would explain the presence of the EPS as well as the thinner cell wall, characteristic of the *K. phaffii* production strains. Also, EPS formation could be related to the missing or altered regulatory function of Hoc1, leading to uncontrolled mannose polymerisation.

### Strategies for exopolysaccharide removal

Different approaches for EPS avoidance could be pursued. Primarily, the function of the Hoc1 protein must be further investigated to engineer novel strains combining the thin cell wall phenotype for efficient transformation and protein secretion with the suppression of EPS formation. In *Vibrio natriegens* EPS byproduct formation could be halved through deletion of a single gene cluster [9]. Moreover, the ancestral *K. phaffii* strain Y-7556, which exhibits lower EPS formation, could be subjected to modifications aimed at enhancing cell wall permeability. Various targets for modifications have been discussed elsewhere [35, 36].

On a process level, employing mannosidases during fermentation processes presents a viable option for EPS degradation. Here, several viable products are available [37]. To circumvent the financial burden associated with enzyme acquisition, the mannosidases could be directly produced by *K. phaffii* [38–43]. However, it is noteworthy that concurrent expression of mannosidases alongside the target protein may entail a reduction in productivity [44]. Moreover, downstream processing necessitates the separation of the enzymes from the target protein, introducing additional complexities. Alternatively, an approach focusing on efficient downstream processing offers a solution for EPS management. Employing diverse chromatography techniques [14, 15] and protein precipitation methods utilizing ammonium sulfate [7], acetone [4] or ethanol [45] can facilitate EPS removal. Furthermore, depending on the recombinant protein size, a filtration step could be considered [46, 47].

Nonetheless, implementation of either strategy entails a substantial increase in process costs. Therefore, the complete avoidance of EPS formation should be addressed at the strain level to maximize energy efficiency and minimize production costs.

## Conclusions

*Komagataella phaffii* secretes a polysaccharide extracellularly. This exopolysaccharide (EPS) formation is revealed to be independent of the integration of protein expression cassettes, methanol induction and carbon source selection, instead directly linked to the strain used. The amount of EPS formed correlates well with the cell mass in the cultivation. The relative EPS concentration is 0.04 g_EPS_/g_CDW_. In high cell density processes, the EPS concentration can reach substantial levels, up to 8.7 g/L in the supernatant, thus, careful consideration in biotechnological applications is mandatory. The EPS presents a challenge in downstream processes for protein purification, necessitating additional processing steps to separate it from the target protein. This contributes to increased downstream costs and operational complexity to obtain high purity protein products. Ignoring EPS formation leads to high impurity of the final product. Further, EPS formation must reduce the yield of target protein on substrate.

There is strong evidence, that in hyper protein producing *K. phaffii* strains the common mutation in the *HOC1* gene is responsible for the high EPS formation. The fermentation of a wildtype strain, lacking this characteristic mutation resulted in a remarkable 76% reduction of free polysaccharide in the supernatant. This highlights the pivotal role of the *HOC1* gene mutation on the EPS formation. Therefore, significant EPS formation will most probably appear in most common *K. phaffii* protein secretion systems originating from the *K. phaffii* NRRL Y-11430 strain, although it often goes unnoticed, due to the lack of signals during PAGE or HPLC–UV.

In conclusion, this work highlights a critical yet overlooked aspect of *K. phaffii* fermentations, the presence and quantification of an EPS impurity, emphasizing the necessity of monitoring and addressing EPS in biotechnological applications, particularly those requiring cost-effective downstream processes. The identification of the *HOC1* gene mutation as a key determinant allows for optimizing *K. phaffii* strains for protein production processes and ultimately enhancing recombinant protein production efficiency and economic viability in biotechnological applications.

### Supplementary Information

**Additional file 1: Figure S1.** Overview of *Pichia pastoris *strain genealogy. **Figure S2.** Determination of starch concentration with the acid hydrolysis method from Figure 1. **Figure S3.** Data for the correlation between OD and CDW for *P. pastoris*. **Figure S4.** Fed-Batch fermentation of *Komagataella phaffii *recombinant protein producing strain I. **Figure S5.** Cell lysis distribution during fermentation. **Figure S6.** Combined exopolysaccharide concentration and optical density for all performed fermentations.

## Data Availability

The datasets used and/or analyzed during the current study are available from the corresponding author on reasonable request.

## Abbreviations

BG11: BSYBG11
CDW: Cell dry weight
EoF: End of fermentation
EPS: Exopolysaccharide
Glu: Glucose
Gly: Glycerol
OD: Optical density
