# Supplementary material for: Understanding exopolysaccharide byproduct formation in Komagataella phaffii fermentation processes for recombinant protein production
Source: Microb Cell Fact. 2024 May 6;23:131. doi: 10.1186/s12934-024-02403-3 (PMC11075191; doi:10.1186/s12934-024-02403-3)
Supplement: Supplementary file 1 — Additional file 1: Figure S1. Overview of Pichia pastoris strain genealogy. Figure S2. Determination of starch concentration with the acid hydrolysis method from Figure 1. Figure S3. Data for the correlation between OD and CDW for P. pastoris. Figure S4. Fed-Batch fermentation of Komagataella phaffii recombinant protein producing strain I. Figure S5. Cell lysis distribution during fermentation. Figure S6. Combined exopolysaccharide concentration and optical density for all performed fermentations. [file 12934_2024_2403_MOESM1_ESM.pdf]

Additional file 1

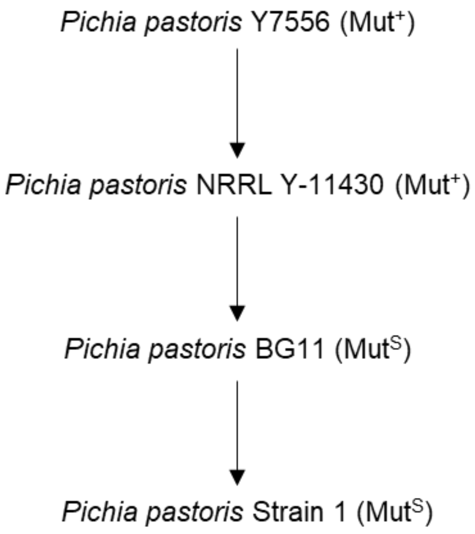

Figure S1: Overview of *Pichia pastoris* strain genealogy (Van Herpe et al., 2022)

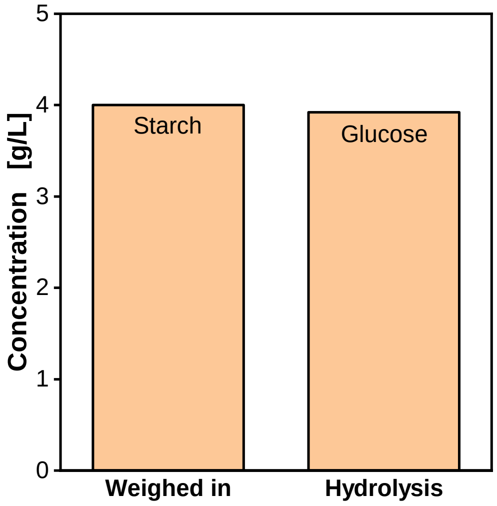

Figure S2: Determination of starch concentration with the acid hydrolysis method from Figure 1. Starch concentration weighed in is compared to glucose concentration determined via HPLC after acid hydrolysis.

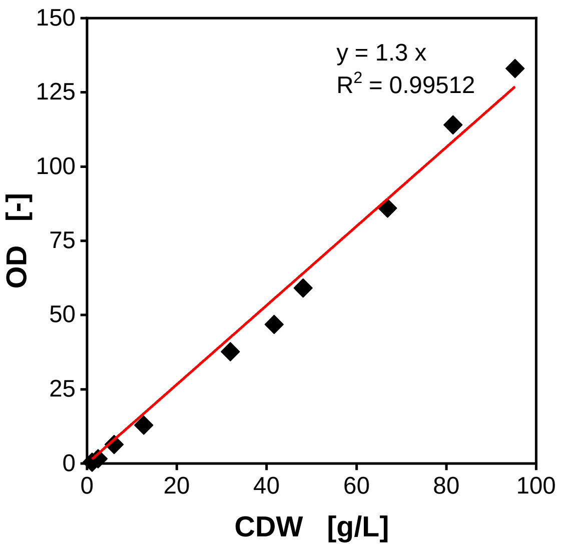

Figure S3: Data for the correlation between OD and CDW for *P. pastoris*. Fit function for data given in red. OD = 1.3·CDW with R^2 = 0.995.

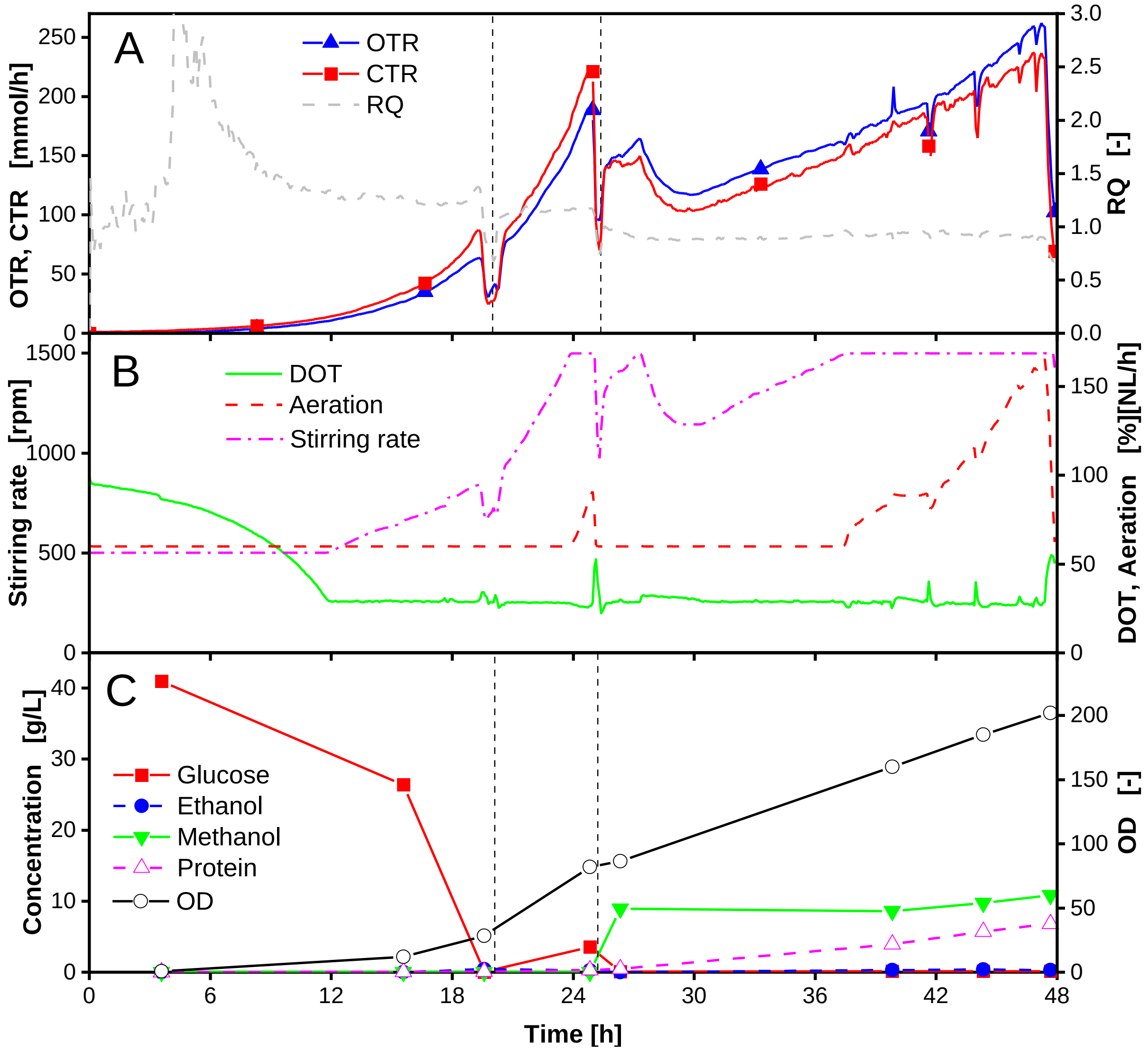

Figure S4: Fed-Batch fermentation of *Komagataella phaffii* recombinant protein producing strain I performed in a 2L stirred tank reactor. In A oxygen transfer rate (OTR), carbon dioxide transfer rate (CTR) and respiratory quotient (RQ) is depicted continuously over time. In B dissolved oxygen tension (DOT), aeration and stirring rate is depicted continuously over time. In C glucose, ethanol, methanol and protein concentration and optical density (OD) is shown measured at sample points. Cultivation was performed with 40 g/L initial glucose concentration. Feeding solution consisted of 650 g/L glucose. Production was induced with 1 vol.% MeOH. 35 mL/L MeOH was added to the feeding solution after induction. Feeding rate F_1 = 11 mL/h *exp(0.2 h^{-1} * t) from 20 h - 25 h. Feeding rate F_2 = 7.5 mL/h *exp(0.05 h^{-1} * t) from 25 h - 48 h. DOT was controlled >30% by cascade control of stirring rate (500-1500 rpm) and aeration rate (60-180 sL/h)

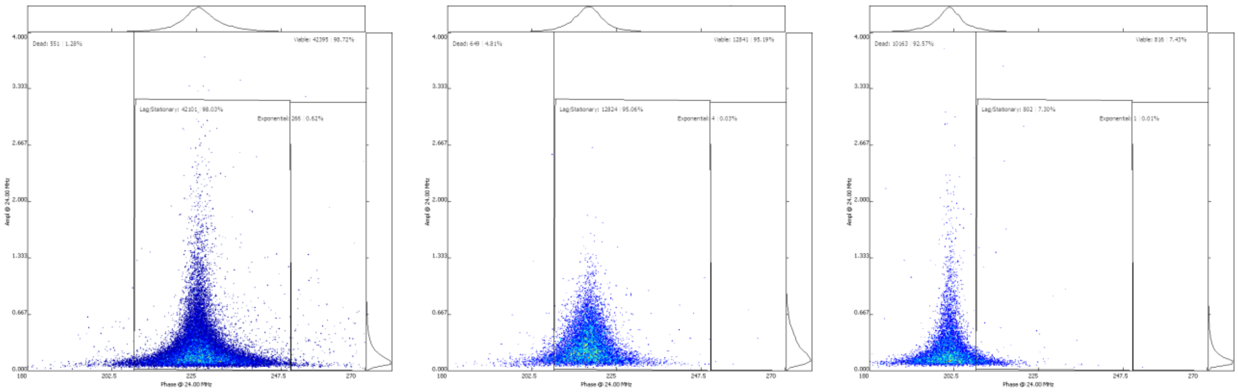

Induction – 1.28% EoF – 4.81% Lysis sample – 92.6%

Figure S5: Cell lysis distribution during fermentation. Samples at induction time and at the end of fermentation (EoF) for process shown in Figure 3 are compared. Cells lysis determined by impedance flow cytometry with an Amphasys device at a frequency of 24 MHz. Samples were diluted in PBS buffer. Cell samples lysed for 20 min at 100 °C were used as positive reference for cell lysis (cell lysis 92.6%).

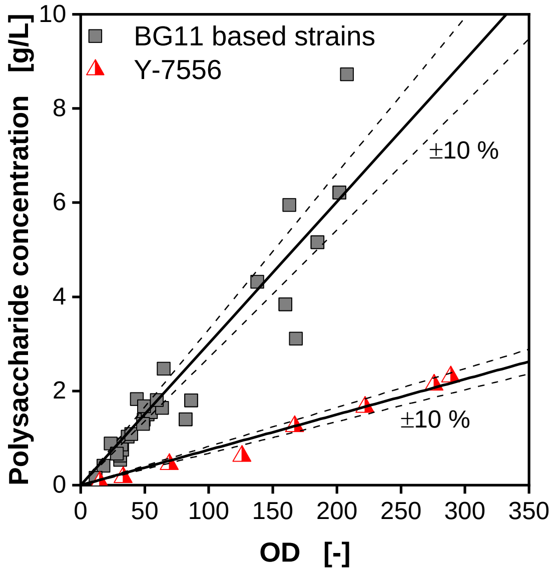

Figure S6: Combined exopolysaccharide concentration and optical density for all performed fermentations. Stirred tank fermentations performed with strains based on the genetic background of the strain BG11 (Strain I and BG11) are shown in grey. Fermentations performed with the strain Y-7556 are shown in red. Correlation and 10% deviation for EPS concentration and OD is shown.
